# What Is the Link between Attention-Deficit/Hyperactivity Disorder (ADHD) and Dyslipidemia in Adults? A German Retrospective Cohort Study

**DOI:** 10.3390/jcm13154460

**Published:** 2024-07-30

**Authors:** Sarah Krieg, Marcel Konrad, Andreas Krieg, Karel Kostev

**Affiliations:** 1 Department of Inclusive Medicine, University Hospital Ostwestfalen-Lippe, Bielefeld University, 33617 Bielefeld, Germany; 2 Health & Social, FOM University of Applied Sciences for Economics and Management, 60486 Frankfurt am Main, Germany; marcel.konrad@fom.de; 3 Department of General and Visceral Surgery, Thoracic Surgery and Proctology, University Hospital Herford, Medical Campus OWL, Ruhr University Bochum, 32049 Herford, Germany; andreas.krieg@klinikum-herford.de; 4 Epidemiology, IQVIA, 60549 Frankfurt, Germany; karel.kostev@iqvia.com

**Keywords:** attention-deficit/hyperactivity disorder, adult ADHD, lipid metabolism disorder, lipid profile, epidemiology, Germany

## Abstract

**Background:** Alterations in the serum lipid profile have been suspected in many psychiatric disorders, such as schizophrenia and depression. However, studies on lipid status in attention-deficit/hyperactivity disorder (ADHD) are sparse and inconsistent. **Methods:** Using the nationwide, population-based IQVIA Disease Analyzer database, this retrospective cohort study included 5367 outpatients from general practices in Germany aged ≥18 years with a documented first diagnosis of ADHD between January 2005 and December 2021 and 26,835 propensity score-matched individuals without ADHD. Study outcomes were the first diagnosis of lipid metabolism disorders as a function of ADHD within up to 10 years of the index date. The cumulative 10-year incidence was analyzed using Kaplan–Meier curves and compared using the log-rank test. In addition, univariate Cox regression analyses were performed. **Results:** In the regression analysis, there was no significant association between ADHD and subsequent lipid metabolism disorders in the total population (HR: 0.94; 95% CI: 0.83–1.08), among women (HR: 1.04; 95% CI: 0.84–1.28), and among men (HR: 0.89; 95% CI: 0.74–1.06). In addition, no significant association was observed in the disease-stratified analyses. **Conclusions:** The findings of this study indicate that ADHD does not exert an influence on lipid metabolism. However, further investigation is warranted, particularly with respect to pharmacological interventions.

## 1. Purpose

Attention-deficit/hyperactivity disorder (ADHD) is one of the most common mental disorders in childhood and adolescence, often persisting into adulthood and is associated with functional deficits and significant health and economic problems. The global prevalence in childhood and adolescence is approximately 5%, with no significant international differences [1,2,3,4], and is reported to be 2.5% in adulthood [5]. According to the diagnostic criteria of the World Health Organization’s (WHO) ICD-10 classification system, a diagnosis of ADHD requires a persistent pattern of inattention, impulsivity, and hyperactivity that interferes with functioning or development. Typically, ADHD symptoms are present to an extent that is inappropriate for the person’s age and developmental stage, occur in a variety of situations, and cause significant distress and/or limitations in family, social, school, or work life. The pathogenesis of ADHD appears to be complex and not well understood. A number of interacting factors are thought to play a role in its development. In particular, genetic predisposition and pre-, peri-, and early postnatal environmental exposures that affect the structural and functional development of the brain are thought to be important factors [6,7,8,9]. ADHD is rarely diagnosed as an isolated disorder. Rather, up to 85% of ADHD patients have an additional comorbid mental disorder [10], and 60% of cases have multiple comorbidities [11,12].

The co-occurring disorders vary with age in the following ways: in early childhood, the focus is on social behavior disorders but also on learning and achievement disorders, especially reading and spelling difficulties, autism spectrum disorders, and developmental coordination disorders; in early adolescence, the focus is on anxiety disorders, depression, and tic disorders. In young adulthood and later adolescence, substance abuse, affective disorders, and personality disorders dominate [11,13,14]. For all comorbid disorders, it is important to distinguish whether the condition is a true comorbidity, a consequence of ADHD, or a subtype of ADHD. However, only a few studies have examined the prevalence of comorbid disorders in adulthood by age and gender.

The treatment of ADHD requires a multimodal therapeutic approach that aims to reduce the immediate symptoms, treat comorbidities, improve social integration, e.g., in school, work, and family, and improve quality of life [15]. In addition to psychoeducation, behavioral therapy, and pharmacological therapy as established pillars of treatment, treatment options such as diets or substitution therapies are being discussed as alternatives and supportive measures [11,16]. Several studies have investigated omega-3 supplementation and suggested a clinically relevant change in lipid status in ADHD, although convincing empirical evidence is still lacking [17,18].

Lipid alterations have been implicated in other psychiatric disorders such as schizophrenia and depression. In ADHD, however, studies on lipid status and changes in serum lipids are sparse and inconsistent [19,20,21,22]. In the absence of comparable previous data, the present retrospective cohort study aims to compare the lipid profile of adult patients with ADHD to adult patients without ADHD using the representative nationwide, population-based IQVIA Disease Analyzer database.

## 2. Methods

### 2.1. Database

This retrospective cohort study was based on routine data from the Disease Analyzer database (IQVIA, Frankfurt, Germany), which contains drug prescriptions, diagnoses, and basic medical and demographic data of outpatients obtained directly and anonymously from the computer systems of general practitioners and specialists [23]. The database includes approximately 3000 office-based physicians in Germany. The panel of practices included in the Disease Analyzer database has previously been shown to be representative of general and specialist practices in Germany [23]. Finally, this database has already been used in a previous study focusing on lipid metabolism disorders [24].

### 2.2. Study Population

**Figure 1:**
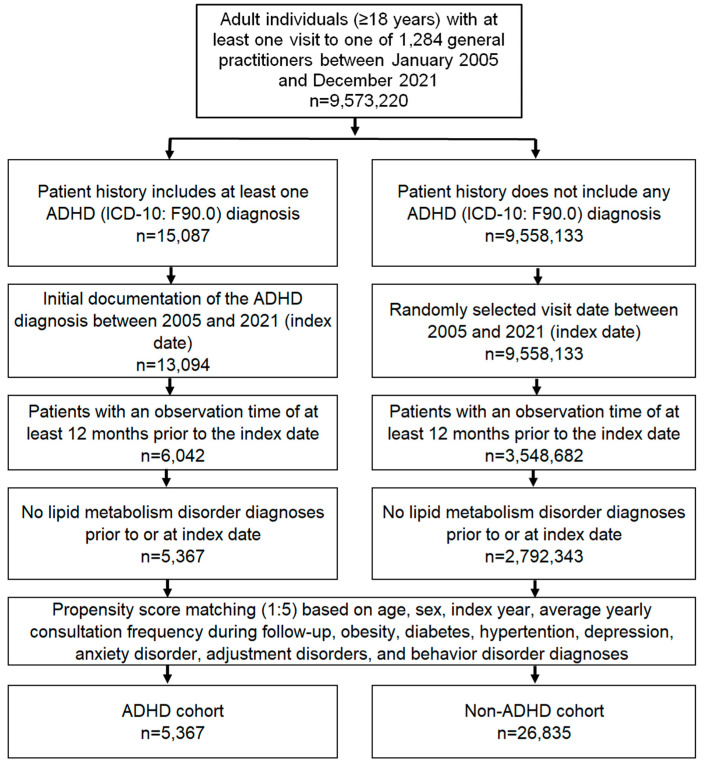
Selection of study patients.

This study included adult patients (≥18 years) with a first documented diagnosis of ADHD (ICD-10: F90.0) in 1284 general practices in Germany between January 2005 and December 2021 (index date; Figure 1). Only patients with an observation period of at least 12 months prior to the index date were included in order to have access to co-diagnoses documented within 12 months prior to the index date. Patients with a diagnosis of lipid metabolism disorders (ICD-10: E78) before or on the index date were excluded.

After applying similar inclusion criteria, individuals without ADHD were matched to patients with ADHD using nearest neighbor propensity score matching (1:5) based on age, sex, index year, average annual visit frequency during follow-up, and co-diagnoses of obesity (ICD-10: E66), diabetes mellitus (ICD-10: E10–E14), and hypertension (ICD-10: I10), depression (ICD-10: F32, F33), anxiety disorders (ICD-10: F41), reaction to severe stress and adjustment disorders (ICD-10: F43), and disorders of adult personality and behavior (ICD-10: F60–F69) documented within 12 months before or on the index date. For the non-ADHD cohort, the index date was a randomly selected visit between January 2005 and December 2021 (Figure 1).

### 2.3. Study Outcomes

The outcomes of this study were as follows: total first diagnoses of lipid metabolism disorders (ICD-10: E78) and pure hypercholesterolemia (ICD-10: E78.0); pure hyperglyceridemia (ICD-10: E78.1); mixed hyperlipidemia (ICD-10: E78.2); and hyperlipidemia, unspecified (ICD-10: E78.5) in the 10 years after the index date as a function of ADHD. Other disorders of lipid metabolism (i.e., hyperchylomicronemia, lipoprotein deficiency, and disorders of bile acid and cholesterol metabolism) were very rarely documented and could not be analyzed separately.

### 2.4. Statistical Analyses

Differences in sample characteristics and diagnosis prevalence between the ADHD and non-ADHD cohorts were compared using the Wilcoxon signed-rank test for continuous variables, the McNemar test for categorical variables with two categories, and the Stuart–Maxwell test for categorical variables with more than two categories. These tests are considered appropriate for paired variables.

The 10-year cumulative incidence of lipid metabolism disorders in total and in defined lipid metabolism disorder types was further examined using Kaplan–Meier curves, and these curves were compared using the log-rank test. Finally, univariable Cox regression analysis was performed to assess the association between ADHD and lipid metabolism disorders. These models were performed separately for female and male subjects. In addition, prescription of ADHD-related medications (methylphenidate, atomoxetine, dexamphetamine, lisdexamfetamine, and guanfacine) was included in the model to evaluate the association between ADHD therapy and lipid metabolism disorders in patients with ADHD only. Results of the Cox regression model are presented as hazard ratios (HRs) and 95% confidence intervals (CIs). A *p*-value of <0.01 was considered statistically significant due to multiple comparisons. Analyses were performed with SAS version 9.4 (SAS Institute, Cary, NC, USA).

## 3. Results

### 3.1. Basic Characteristics of the Study Sample

The present study included 5367 individuals with and 26,835 without ADHD. The basic characteristics of study patients are displayed in Table 1. Median age was 29 (interquartile range (IQR): 18) years; 63.3% were male. Patients visited physicians in median five times per year during the follow-up. Predefined comorbidities were not frequent (7.0% with obesity, 4.1% with hypertension, and 3.6% with diabetes). Moreover, 1073 (20.0%) of ADHD patients received at least one prescription of relevant drug during the study period.

### 3.2. Cumulative Incidence of Lipid Metabolism Disorders among Patients with and without ADHD

**Figure 2:**
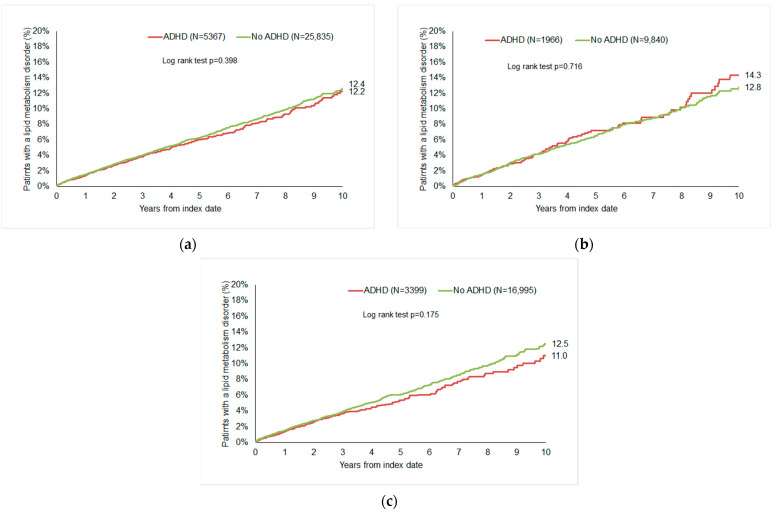
Cumulative 10-year incidence of lipid metabolism disorders in patients with and without ADHD. (**a**) All patients; (**b**) women; and (**c**) men.

After up to ten years of follow-up, 12.2% of ADHD patients and 12.4% of non-ADHD patients were diagnosed with lipid metabolism disorders (*p* = 0.398, Figure 2a). There were 14.3% of women with ADHD and 12.8% of women without ADHD (*p* = 0.716), as well as 11.0% vs. 12.5% of men (*p* = 0.175), with a diagnosis of lipid metabolism disorders.

### 3.3. Association of ADHD with Lipid Metabolism Disorders Diagnoses

In the regression analysis, there was no significant association between ADHD and subsequent lipid metabolism disorders in the total population (HR: 0.94; 95% CI: 0.83–1.08), among women (HR: 1.04; 95% CI: 0.84–1.28), and among men (HR: 0.89; 95% CI: 0.74–1.06). In the disease-stratified analyses, no significant associations were observed either (Table 2).

### 3.4. Role of ADHD Therapy

In an additional regression model, no association was seen between ADHD therapy and subsequent lipid metabolism disorders in the total population (HR: 0.82; 95% 0.60–1.13), women (HR: 0.57; 95% 0.33–1.01), and men (HR: 1.01; 95% 0.69–1.49). Also, in disease-stratified analyses, no significant associations were observed.

## 4. Discussion

In this retrospective cohort study, a large cohort of 5367 adult outpatients with ADHD was analyzed for lipid metabolism disorders using the representative database. Data from adults aged 18 years and older with ADHD were compared with a cohort of propensity score-matched individuals without ADHD.

Overall, the findings of this study revealed no significant association between ADHD and lipid metabolism disorders in the overall study population, which included both female and male patients. Additionally, this study found no evidence of a link between ADHD and specific lipid metabolism disorders, such as pure hypercholesterolemia, pure hyperglyceridemia, and mixed hyperlipidemia. Furthermore, an additional regression model indicated that ADHD medication treatment was not associated with subsequent lipid disorders.

There is a growing interest in the role of lipids in the development and maintenance of psychiatric disorders [25,26,27,28]. For example, studies have implicated lipid metabolism in depression [26], Alzheimer’s disease [27], and schizophrenia [28]. Niemann–Pick type C disease, an autosomal recessive lysosomal storage disorder, is a severe form of impaired cholesterol metabolism caused by a mutation, usually in the NPC-1 gene (Niemann–Pick type C1 gene). In this disorder, defective intracellular cholesterol transport mechanisms lead to cholesterol accumulation, resulting in severe cognitive impairment and reduced life expectancy [29]. Against this background, a post hoc analysis published in 2019 by Pinho et al. compared serum levels of total cholesterol, high-density lipoprotein (HDL), low-density lipoprotein (LDL), and triglycerides in patients with ADHD and controls without ADHD using data from the nationwide population-based German Health Interview and Examination Survey for Children and Adolescents (KiGGS) of the Robert Koch Institute (RKI). Using multivariate and univariate models, the authors showed small but statistically significant associations between total cholesterol, high-density lipoprotein (HDL), low-density lipoprotein (LDL), and triglycerides in children with ADHD compared with controls. However, serum triglyceride concentrations were higher and serum LDL concentrations were lower in ADHD patients [19]. These findings contrast with a 2018 study by Avcil et al., in which 32 boys with ADHD were compared with a control group of 29 healthy subjects for their lipid profile. They concluded that the average levels of triglycerides (TGs), low-density lipoprotein cholesterol (LDL-C), and high-density lipoprotein cholesterol (HDL-C) were significantly lower in the ADHD group than in the control group [20]. On the other hand, Ugur et al. reported higher cholesterol and LDL levels in a total sample of 88 children aged 8 to 12 years with ADHD compared to 88 healthy children. Despite controlling for age, gender, and body mass index (BMI), the authors found that total cholesterol and LDL levels were significantly higher in the ADHD group than in the healthy control group, whereas TG and HDL cholesterol levels were similar in both groups [21]. In contrast, in a small study of nine boys with ADHD compared with 11 controls, Irmisch et al. found significantly higher serum HDL concentrations [22]. These very heterogeneous and partly contradictory results underline the importance of further research in this area, not only with regard to a possible explanation of the pathogenesis but also with regard to a possible alteration of the lipid profile with child development, as well as possible treatment options and prevention approaches. Interestingly, several studies have investigated the effect of fatty acid supplements on ADHD. In a meta-analysis of nine different studies, Hawkey and Nigg showed that all 586 children with ADHD had low blood levels of omega-3 fatty acids and that supplementation could slightly improve symptoms [18]. In contrast, Agostoni et al. found only a weakly significant effect that was mostly limited to one dimension, either hyperactivity or inattention [30]. However, studies are heterogeneous, as different fatty acids were substituted or administered in addition to medication, so that effects cannot always be clearly attributed to fatty acid supplementation [30]. Based on this, the German S3 Guideline on ADHD does not yet recommend substitution, and there is a need for further research in this area [31]. Additionally, further research is needed to better understand the role of fatty acids in ADHD. Despite the absence of an association between lipid metabolism disorders and ADHD in our study, an intriguing yet under-researched hypothesis regarding an underlying pathophysiological mechanism between other psychiatric disorders and lipid metabolism disorders is being discussed. This hypothesis, known as the membrane hypothesis, postulates that alterations in the composition and structure of cell membranes may play a role in the pathogenesis of psychiatric disorders [32]. In this context, animal studies have shown that the membrane properties of the cell, such as fluidity, viscosity, and functionality, are strongly dependent on the lipid composition [32]. In addition, it has been shown that a reduced cholesterol content in neuronal membranes can lead to a reduced number of serotonin receptors and reduced impulse control [32]. Alterations in the lipid composition of neuronal membranes may play an important role in the neuronal transmission of stimuli and thus in the pathogenesis of disturbed neuronal control circuits in ADHD. Interestingly, Dietschy et al. have shown that central and peripheral cholesterol metabolism appear to be separate, as the blood–brain barrier itself is impermeable to cholesterol [33]. The hypothesis of cholesterol transfer across the blood–brain barrier was also refuted by Castellanos et al. in animal experiments [34], and it is suggested that most cholesterol in the central nervous system (CNS) is synthesized de novo intracerebrally [35,36]. In contrast, Meijer et al. found differences in methylation of the cholesterol signaling genes APOB (apolipoprotein B gene) and LPAR5 (lysophosphatidic acid receptor 5 gene) in blood samples from individuals with a persistent or remitting ADHD diagnosis, although conclusions about central nervous mechanisms should be treated with caution [37]. In conclusion, basic research findings are not yet sufficient to be translated into clinical practice. The need for further research in this area is therefore even greater in order to develop a new therapeutic approach in the future or to take preventive measures at an early stage of diagnosis.

Although our study did not find an association between medication for ADHD and subsequent lipid metabolism disorders, other studies in this area have suggested that many psychotropic medications may even have a beneficial effect on the lipid profile in blood plasma and its metabolism. In a small study of 42 patients with an average age of 16 years who were treated for ADHD in adolescent psychiatric facilities between 2003 and 2007, Charach et al. investigated the influence of the piperidine derivative methylphenidate on the plasma lipid profile of patients diagnosed with ADHD. Blood samples were analyzed for total cholesterol, LDL-C, HDL-C, triglycerides, apolipoprotein A, apolipoprotein B, and lipoprotein (a) (Lp(a)) before the start of treatment and after 3 months of continuous treatment. The results showed that methylphenidate improved the lipid profile by significantly lowering total cholesterol, triglycerides, LDL-C, and Lp(a) [38]. Again, further studies, especially in a large cohort of ADHD patients, are needed to clarify these findings.

Finally, the present study is limited by several unavoidable aspects, mainly related to the study design and methodology, that have already been described in detail in the past [39]. First, all diagnoses were coded with ICD-10 codes, which may lead to misclassification or undercoding of certain diagnoses. Additionally, due to the relatively small number of ADHD cases, 1:5 matching was used for analysis. Although age and sex are included in the analyses, information on patients’ lifestyle (e.g., dietary habits and physical activity) and other socio-demographic characteristics such as occupation, social class, and income are missing. Furthermore, clinical parameters, in particular blood test results, questionnaire results, and clinical symptoms, were documented only for a small part of patients. Although we also examined the influence of ADHD medication on the occurrence of lipid metabolism disorders, we have no information on other concomitant medications that may have influenced lipid and lipoprotein metabolism. Further studies are required to elucidate the extent to which statin therapy, in particular, which is often employed to treat hyperlipidemia, especially hypercholesterolemia, can influence the occurrence of ADHD.

The Disease Analyzer database is lacking in data pertaining to socioeconomic status, including patients’ education and income, and lifestyle-related risk factors, such as smoking, alcohol consumption, and physical activity. Consequently, these variables could not be included in this study. Moreover, the database is deficient in data pertaining to mortality quality that would be suitable for the purposes of this study. The specific type of ADHD was not available, as this information has not been documented by general practitioners. A further limitation is the absence of data on the duration of ADHD. The initial ADHD diagnosis by a general practitioner was considered the index data in this study. Nevertheless, it is possible that some patients may have been diagnosed with ADHD during childhood and that the cohort profile may differ between incident and prevalent ADHD patients. This may also be a confounding factor in the relationship between ADHD and lipid metabolism disorders.

Finally, our study does not allow causal attribution, only associations. However, it must be emphasized that our study is one of the few to examine the association between lipid alterations in patients with ADHD, especially in adulthood. Finally, the IQVIA Disease Analyzer database used for the analyses in this study is representative of the general population and has been extensively published [40,41,42] and shown to be valid [23].

## 5. Conclusions

Overall, our study adds to the literature and provides some evidence that ADHD is not associated with disorders of lipid metabolism. However, the heterogeneous and partly contradictory results make it clear how important further research in this area is, not only with regard to a possible explanation of the pathogenesis but also with regard to a possible change in lipid levels with individual development as well as possible treatment options and prevention approaches.

## Figures and Tables

**Table 1:** Baseline characteristics of the study sample (after 1:5 propensity score matching).

Variable	Proportion among Patients with ADHD (N, %) N = 5367	Proportion among Patients without ADHD (N, %) N = 26,835	*p*-Value
Age (Median, IQR)	29 (18)	29 (18)	0.516
Age 18–20	1037 (19.3)	4995 (18.6)	0.636
Age 21–30	1902 (35.4)	9592 (35.7)	
Age 31–40	1112 (20.7)	5642 (21.0)	
Age 41–50	758 (14.1)	3705 (13.8)	
Age >50	558 (10.4)	2901 (10.8)	
Female	1966 (36.7)	9840 (36.7)	1.000
Male	3399 (63.3)	16,995 (63.3)	
Number of physician visits per year during the follow-up (Median, IQR)	5 (7)	5 (7)	0.942
Obesity	374 (7.0)	1756 (6.5)	0.253
Diabetes	195 (3.6)	965 (3.6)	0.894
Hypertension	594 (11.1)	2811 (10.5)	0.198
Depression	2038 (38.0)	10,287 (38.3)	0.619
Anxiety disorders	824 (15.4)	4132 (15.4)	0.934
Reaction to severe stress and adjustment disorders	1086 (20.2)	5414 (20.2)	0.921
Disorders of adult personality and behavior	613 (11.4)	3005 (11.2)	0.541

Proportions of patients in N, % given, unless otherwise indicated. SD: standard deviation. IQR: interquartile range.

**Table 2:** Association between ADHD and subsequent lipid metabolism disorders in patients followed in general practices in Germany (univariable Cox regression models).

	All Patients		Women		Men	
Outcome Diagnosis	HR (95% CI)	*p*-Value	HR (95% CI)	*p*-Value	HR (95% CI)	*p*-Value
Lipid metabolism disorders (total)	0.94 (0.83–1.08)	0.398	1.04 (0.84–1.28)	0.713	0.89 (0.74–1.06)	0.175
Pure hypercholesterolemia	1.00 (0.83–1.21)	0.976	1.18 (0.90–1.55)	0.221	0.87 (0.66–1.14)	0.313
Pure hyperglyceridemia	1.00 (0.65–1.56)	0.995	0.50 (0.15–1.67)	0.262	1.14 (0.71–1.84)	0.582
Mixed hyperlipidemia	1.16 (0.73–1.84)	0.544	1.43 (0.64–3.20)	0.386	1.05 (0.59–1.86)	0.870
Hyperlipidemia, unspecified	0.89 (0.69–1.16)	0.386	1.03 (0.68–1.56)	0.882	0.81 (0.58–1.14)	0.234

## Data Availability

The data that support the findings of this study are available from the corresponding author upon reasonable request.

## References

[1] Faraone S.V., Asherson P., Banaschewski T., Biederman J., Buitelaar J.K., Ramos-Quiroga J.A., Rohde L.A., Sonuga-Barke E.J., Tannock R., Franke B. (2015). Attention-deficit/hyperactivity disorder. Nat. Rev. Dis. Primers.

[2] Polanczyk G., de Lima M.S., Horta B.L., Biederman J., Rohde L.A. (2007). The worldwide prevalence of ADHD: A systematic review and metaregression analysis. Am. J. Psychiatry.

[3] Polanczyk G.V., Willcutt E.G., Salum G.A., Kieling C., Rohde L.A. (2014). ADHD prevalence estimates across three decades: An updated systematic review and meta-regression analysis. Int. J. Epidemiol..

[4] Schlack R., Mauz E., Hebebrand J., Hölling H. (2014). [Has the prevalence of parent-reported diagnosis of attention deficit hyperactivity disorder (ADHD) in Germany increased between 2003–2006 and 2009–2012? Results of the KiGGS-study: First follow-up (KiGGS Wave 1)]. Bundesgesundheitsblatt Gesundheitsforschung Gesundheitsschutz.

[5] Simon V., Czobor P., Bálint S., Mészáros A., Bitter I. (2009). Prevalence and correlates of adult attention-deficit hyperactivity disorder: Meta-analysis. Br. J. Psychiatry J. Ment. Sci..

[6] Smith T.F. (2010). Meta-analysis of the heterogeneity in association of DRD4 7-repeat allele and AD/HD: Stronger association with AD/HD combined type. Am. J. Med. Genet. Part B Neuropsychiatr. Genet. Off. Publ. Int. Soc. Psychiatr. Genet..

[7] Li D., Sham P.C., Owen M.J., He L. (2006). Meta-analysis shows significant association between dopamine system genes and attention deficit hyperactivity disorder (ADHD). Hum. Mol. Genet..

[8] Castellanos F.X., Tannock R. (2002). Neuroscience of attention-deficit/hyperactivity disorder: The search for endophenotypes. Nat. Rev. Neurosci..

[9] Pauli-Pott U., Dalir S., Mingebach T., Roller A., Becker K. (2013). Do different ADHD-related etiological risks involve specific neuropsychological pathways? An analysis of mediation processes by inhibitory control and delay aversion. J. Child Psychol. Psychiatry Allied Discip..

[10] Pliszka S.R. (1998). Comorbidity of attention-deficit/hyperactivity disorder with psychiatric disorder: An overview. J. Clin. Psychiatry.

[11] Jensen C.M., Steinhausen H.C. (2015). Comorbid mental disorders in children and adolescents with attention-deficit/hyperactivity disorder in a large nationwide study. Atten. Deficit Hyperact. Disord..

[12] Steinhausen H.C. (2010). Komorbiditäten und assoziierte Probleme. Grundlagen, Klinik, Therapie und Verlauf der Aufmerksamkeitsdefizit-Hyperaktivitätsstörung.

[13] Steinhausen H.C., Holtmann M., Banaschewski T., Steinhausen H., Döpfner M., Holtmann M., Philipsen A., Rothenberger A. (2020). Komorbiditäten und assoziierte Probleme im Kindes-und Jugendalter. Handbuch ADHS: Grundlagen, Klinik, Therapie und Verlauf der Aufmerksamkeitsdefizit-Hyperaktivitätsstörung.

[14] Matthies S., Lam A., Philipsen A., Steinhausen H.C., Döpfner M., Holtmann M., Philipsen A., Rothenberger A. (2020). Komorbide Störungen im Erwachsenenalter. Handbuch ADHS: Grundlagen, Klinik, Therapie und Verlauf der Aufmerksamkeitsdefizit-Hyperaktivitätsstörung.

[15] Steinhausen H.C., Döpfner M., Holtmann M., Philipsen A., Rothenberger A., Steinhausen H.C., Döpfner M., Holtmann M., Philipsen A., Rothenberger A. (2020). Therapien-Einleitung und Überblick. Handbuch ADHS: Grundlagen, Klinik, Therapie und Verlauf der Aufmerksamkeitsdefizit-Hyperaktivitätsstörung.

[16] Banaschewski T., Becker K., Döpfner M., Holtmann M., Rösler M., Romanos M. (2017). Attention-Deficit/Hyperactivity Disorder. Dtsch. Arztebl. Int..

[17] Bloch M.H., Qawasmi A. (2011). Omega-3 fatty acid supplementation for the treatment of children with attention-deficit/hyperactivity disorder symptomatology: Systematic review and meta-analysis. J. Am. Acad. Child Adolesc. Psychiatry.

[18] Hawkey E., Nigg J.T. (2014). Omega-3 fatty acid and ADHD: Blood level analysis and meta-analytic extension of supplementation trials. Clin. Psychol. Rev..

[19] Pinho R., Wang B., Becker A., Rothenberger A., Outeiro T.F., Herrmann-Lingen C., Meyer T. (2019). Attention-deficit/hyperactivity disorder is associated with reduced levels of serum low-density lipoprotein cholesterol in adolescents. Data from the population-based German KiGGS study. World J. Biol. Psychiatry Off. J. World Fed. Soc. Biol. Psychiatry.

[20] Avcil S. (2018). Association between altered lipid profiles and attention deficit hyperactivity disorder in boys. Nord. J. Psychiatry.

[21] Ugur C., Uneri O.S., Goker Z., Sekmen E., Aydemir H., Solmaz E. (2018). The assessment of serum lipid profiles of children with attention deficit hyperactivity disorder. Psychiatry Res..

[22] Irmisch G., Thome J., Reis O., Hässler F., Weirich S. (2011). Modified magnesium and lipoproteins in children with attention deficit hyperactivity disorder (ADHD). World J. Biol. Psychiatry Off. J. World Fed. Soc. Biol. Psychiatry.

[23] Rathmann W., Bongaerts B., Carius H.J., Kruppert S., Kostev K. (2018). Basic characteristics and representativeness of the German Disease Analyzer database. Int. J. Clin. Pharmacol. Ther..

[24] Zingel R., Bohlken J., Riedel-Heller S., Barth S., Kostev K. (2021). Association Between Low-Density Lipoprotein Cholesterol Levels, Statin Use, and Dementia in Patients followed in German General Practices. J. Alzheimer’s Dis. JAD.

[25] Maes M., Smith R., Christophe A., Vandoolaeghe E., Van Gastel A., Neels H., Demedts P., Wauters A., Meltzer H.Y. (1997). Lower serum high-density lipoprotein cholesterol (HDL-C) in major depression and in depressed men with serious suicidal attempts: Relationship with immune-inflammatory markers. Acta Psychiatr. Scand..

[26] Bot M., Milaneschi Y., Al-Shehri T., Amin N., Garmaeva S., Onderwater G.L.J., Pool R., Thesing C.S., Vijfhuizen L.S., Vogelzangs N. (2020). Metabolomics Profile in Depression: A Pooled Analysis of 230 Metabolic Markers in 5283 Cases With Depression and 10,145 Controls. Biol. Psychiatry.

[27] Chew H., Solomon V.A., Fonteh A.N. (2020). Involvement of Lipids in Alzheimer’s Disease Pathology and Potential Therapies. Front. Physiol..

[28] Schneider M., Levant B., Reichel M., Gulbins E., Kornhuber J., Müller C.P. (2017). Lipids in psychiatric disorders and preventive medicine. Neurosci. Biobehav. Rev..

[29] Grau A.J., Weisbrod M., Hund E., Harzer K. (2003). [Niemann-Pick disease type C--a neurometabolic disease through disturbed intracellular lipid transport]. Der Nervenarzt.

[30] Agostoni C., Nobile M., Ciappolino V., Delvecchio G., Tesei A., Turolo S., Crippa A., Mazzocchi A., Altamura C.A., Brambilla P. (2017). The Role of Omega-3 Fatty Acids in Developmental Psychopathology: A Systematic Review on Early Psychosis, Autism, and ADHD. Int. J. Mol. Sci..

[31] Arbeitsgemeinschaft der Wissenschaftlichen Medizinischen Fachgesellschaften (AWMF) (2017). S3 Guideline on ADHD in Children, Adolescents, and Adults. https://www.awmf.org/leitlinien/detail/ll/028-045.html.

[32] Engelberg H. (1992). Low serum cholesterol and suicide. Lancet.

[33] Dietschy J.M., Turley S.D. (2001). Cholesterol metabolism in the brain. Curr. Opin. Lipidol..

[34] Castellanos F.X., Lee P.P., Sharp W., Jeffries N.O., Greenstein D.K., Clasen L.S., Blumenthal J.D., James R.S., Ebens C.L., Walter J.M. (2002). Developmental trajectories of brain volume abnormalities in children and adolescents with attention-deficit/hyperactivity disorder. Jama.

[35] Vance J.E., Hayashi H., Karten B. (2005). Cholesterol homeostasis in neurons and glial cells. Semin. Cell Dev. Biol..

[36] Zhang J., Liu Q. (2015). Cholesterol metabolism and homeostasis in the brain. Protein Cell.

[37] Meijer M., Klein M., Hannon E., van der Meer D., Hartman C., Oosterlaan J., Heslenfeld D., Hoekstra P.J., Buitelaar J., Mill J. (2020). Genome-Wide DNA Methylation Patterns in Persistent Attention-Deficit/Hyperactivity Disorder and in Association With Impulsive and Callous Traits. Front. Genet..

[38] Charach G., Kaysar N., Grosskopf I., Rabinovich A., Weintraub M. (2009). Methylphenidate has positive hypocholesterolemic and hypotriglyceridemic effects: New data. J. Clin. Pharmacol..

[39] Labenz C., Wörns M.A., Adarkwah C.C., Galle P.R., Schattenberg J.M., Kostev K. (2020). Proton pump inhibitors increase risk of bone fractures in men with cirrhosis: A population-based study. Aliment. Pharmacol. Ther..

[40] Huber Y., Labenz C., Michel M., Wörns M.A., Galle P.R., Kostev K., Schattenberg J.M. (2020). Tumor Incidence in Patients with Non-Alcoholic Fatty Liver Disease. Dtsch. Arztebl. Int..

[41] Labenz C., Huber Y., Michel M., Nagel M., Galle P.R., Kostev K., Schattenberg J.M. (2020). Nonalcoholic Fatty Liver Disease Increases the Risk of Anxiety and Depression. Hepatol. Commun..

[42] Jacob L., Smith L., Koyanagi A., Schnitzler A., Il Shin J., Kostev K. (2021). Association between osteoarthritis and the incidence of Parkinson’s disease in the United Kingdom. Clin. Park. Relat. Disord..

